# Integrating Generative AI into public health education: a mixed methods evaluation of student experiences

**DOI:** 10.3389/fpubh.2026.1825937

**Published:** 2026-07-15

**Authors:** Zelalem Mengesha, Jennifer Estellore, Jennifer Smith, Mathew Sunil George, Rosemary A. McFarlane, Jinal Parmar

**Affiliations:** 1 Discipline of Public Health, University of Canberra, Canberra, ACT, Australia; 2 Learning and Teaching, University of Canberra, Canberra, ACT, Australia

**Keywords:** AI literacy, assessment design, generative AI, higher education pedagogy, student perspectives

## Abstract

**Introduction:**

As Generative Artificial Intelligence (GenAI) continues to influence pedagogical practices in higher education, prevailing discourse has largely focused on issues of assessment integrity, often overlooking student perspectives. Yet, understanding student voices is essential to ensure that AI-enhanced teaching and assessment remain student centred, equitable, and aligned with intended learning outcomes. This study aimed to explore postgraduate health science students’ perceptions and experiences of the structured integration of GenAI into teaching and assessment within the Epidemiology and Principles of Research unit at the University of Canberra.

**Methods:**

A mixed-methods study was conducted using pre- and post-intervention surveys administered to all enrolled students, with 78 participants completing the evaluation. The intervention involved the scaffolded integration of GenAI into a critical appraisal assessment. Quantitative data were analysed using descriptive statistics and Fisher’s exact test to assess changes over time. Qualitative data from open-ended responses were analysed using thematic analysis following Braun and Clarke’s six-phase approach.

**Results:**

Students reported statistically significant improvements in their understanding of GenAI and perceived ability to use it effectively (*p* < 0.001). Thematic analysis identified five key themes: tensions between automation and authentic learning; prompt literacy as a new academic skill; GenAI as a support for metacognitive engagement; ethical ambiguity and cognitive dissonance; and future-oriented learning.

**Conclusion:**

The findings highlight the importance of thoughtful pedagogical designs that center student voice, support critical engagement, and prepare learners for responsible and reflective use in future professional practice.

## 1 Introduction

The rapid advancement of Generative Artificial Intelligence (GenAI) technologies has introduced both opportunities and challenges across multiple sectors, including higher education, where emerging tools are increasingly influencing teaching and learning practices (1). Tools such as ChatGPT, Copilot, and Gemini are being used to support how students access, process, and engage with academic content (2). These tools extend beyond simple information retrieval by enabling students to generate structured responses, engage with complex concepts, and receive feedback on their academic work, contributing to more interactive and personalised learning experiences (3). As a result, established approaches to teaching, learning, and assessment are being reconsidered within higher education contexts (4).

With GenAI becoming more prevalent in university settings, educators are increasingly examining its implications for teaching and assessment, particularly in disciplines where critical thinking and originality are central to learning outcomes (5). While integration of GenAI has been associated with opportunities for enhanced accessibility, creativity and personalised learning (6), it also raises concerns related to academic integrity, cognitive engagement and the ethical boundaries of AI-supported outputs (7). Despite growing interest in this area, there remains limited empirical evidence examining how GenAI influences learning processes and outcomes across specific disciplinary contexts, making it difficult to evaluate its educational implications (8).

The implications of GenAI are particularly salient in the context of assessment design, where its use challenges conventional approaches to evaluating student learning (9). As educators consider how to integrate GenAI into curricula in meaningful and ethical ways, several important questions arise regarding how students engage with these tools during assessment tasks (3), how GenAI may influence their learning processes (10), and how its use relates to the development of core disciplinary competencies (11). In public health education, where critical appraisal, evidence-based reasoning, and ethical judgment are foundational competencies, these questions are particularly relevant (12, 13). Students in this field are expected to critically evaluate scientific literature, interpret complex data and apply rigorous methodological frameworks (14). Consequently, concerns have been raised regarding the reliability of AI-generated content, potential changes in student engagement with critical analysis, and implications for academic integrity. These considerations highlight the need for discipline-specific research to guide the ethical and effective integration of GenAI in developing core competencies in public health education (13).

Recent literature has increasingly examined the integration of GenAI tools in higher education, including studies exploring student perceptions and experiences of their use in learning processes (11, 15, 16). Some studies have focused on the use of GenAI in tasks such as writing, coding and information retrieval (12, 16–18), while others have explored its potential role as a learning support tool in health research training (19–21). However, relatively few studies have examined the integration of GenAI in public health and epidemiology education, with existing work primarily focusing on general patterns of use and perceptions of associated risks and benefits among students and academics (12, 22).

In the Australian context, current discourse has predominantly focused on issues related to assessment integrity (23), with comparatively limited attention to the role of student perspectives in informing GenAI-informed teaching and assessment practices (24). There is also a lack of research examining the structured integration of GenAI into discipline-specific assessments (25), such as critical appraisal tasks in epidemiology. Furthermore, the impact of GenAI on student learning experiences within formal assessment contexts has not been systematically investigated. This gap highlights the need for research that captures both students’ experiences and their engagement with GenAI within discipline-specific learning tasks. Addressing this gap, the present study aimed to explore postgraduate health science students’ perceptions and experiences of the structured integration of GenAI into teaching and assessment within a public health education context.

## 2 Methods

### 2.1 GenAI integration into public health education at the University of Canberra

The integration of GenAI into the Epidemiology and Principles of Research unit is grounded in constructivist learning theory, which suggests that learners actively construct knowledge through experience, reflection, and interaction with their environment (26, 27). In the context of this integration, authentic learning is understood as students’ engagement in core disciplinary practices such as searching for, analysing, and critically evaluating academic literature using established scholarly methods. Within the Epidemiology and Principles of Research unit, this includes the application of epidemiological reasoning, appraisal frameworks, and evidence-based analysis. This conceptualisation aligns with constructivist principles, which position learning as an active process of knowledge construction through engagement with disciplinary tools and practices. This approach is particularly relevant in higher education, where fostering inquiry, reflection, and independent thinking is key. Social constructivism, as developed by Vygotsky (47), places further emphasis on the social and cultural contexts of learning, and the role of new tools such as GenAI as mediators of cognitive development (28). The unit design also draws on the principle of constructive alignment (29) to ensure that learning activities and assessment tasks directly align with the unit learning outcomes.

The decision to incorporate GenAI was guided by two key considerations. First, there was a genuine risk that students might use GenAI tools without permission to complete the critical appraisal assessment, worth 40% of the unit’s final grade, potentially breaching academic integrity and undermining the validity of measuring the intended learning outcomes (ILOs). Here, assessment validity refers to ensuring that a task aligns with the capabilities students are expected to demonstrate as set out in the intended learning outcomes (30). Second, when explicitly integrated, GenAI use does not undermine the central role of students in completing the task. Rather, it supports their ability to demonstrate the required knowledge and skills, as long as its use is guided, transparent, and accompanied by critical reflection. The integration process involved a series of critical steps to ensure that GenAI use was effectively aligned with the unit’s ILOs and appropriately scaffolded within the assessment design. Importantly, the GenAI integration’ is not a fixed strategy determined at the outset; rather, it is dynamic and iterative, continuously refined in response to student feedback and emerging insights. The steps outlined below reflect the design of the intervention, each accompanied by a brief rationale.

#### 2.1.1 Step 1: pedagogical alignment

Applying the principles of constructive alignment (29), this step involved a thorough review of the Epidemiology and Principles of Research unit’s ILOs and assessments. The review highlighted two key points. One, the ILOs specify higher-order thinking, specifically analysis and evaluation, based on the revised Bloom’s Taxonomy (31). Two, a detailed deconstruction of the assessment design of the unit’s major critical appraisal assessment task showed that students are required to engage in complex cognitive processes to demonstrate achievement of these outcomes, even when GenAI is integrated. These analyses led to the redesign of the assessment to ensure it remains robust by assessing the appropriate level of complex cognition and maintains its validity by measuring what it purports to assess, as set out in the unit’s learning outcomes.

#### 2.1.2 Step 2: scaffolding

The next priority was to scaffold students’ ability to complete their critical appraisal assessment using GenAI effectively and ethically. For Epidemiology and Principles of Research unit, scaffolding was executed through a series of learning activities in a 2-hour tutorial utilizing both digital and classroom environments. The development of learning activities was informed by Salmon’s (32) five-stage model of online learning, which provides a structured and scaffolded approach to fostering student engagement and skill development. The following section outlines how each stage of Salmon’s model was addressed in the design and delivery of the GenAI integration (intervention).

##### 2.1.2.1 Access and motivation

This stage focuses on equipping students with the technical knowledge to use Copilot efficiently and developing their understanding of GenAI and its relevance in the context of epidemiology and public health. Our pre-intervention survey revealed wide variation in students’ GenAI literacy and prior experience, resulting in diverse levels of confidence and readiness to engage with GenAI tools. To address this, we developed and delivered a dedicated lecture and tutorial that introduced the conceptual, practical, and ethical dimensions of GenAI. The lecture and tutorial activities were deliberately scaffolded to enable meaningful participation for all students regardless of their starting point (33). This approach helped mitigate differences in GenAI literacy that could otherwise impact students’ ability to complete the assessment task requiring GenAI use.

The session began by outlining the rationale for integrating GenAI into epidemiology, highlighting its growing relevance in data analysis, literature synthesis, and evidence-based decision-making in public health. We then introduced students to the basic mechanics of GenAI: what it is, how it is trained, and the types of data sources that underpin its outputs. This was followed by a critical discussion of GenAI’s limitations, including known issues such as algorithmic bias, hallucination, and the generation of factually inaccurate or misleading content. To enhance students’ practical engagement, we introduced cognitive strategies and prompt engineering techniques, guiding them on how to craft effective prompts that support meaningful interaction with GenAI. We also explored the various stages of a research project where GenAI could add value, encouraging students to think critically about when and how to use these tools appropriately. Institutional policies and support resources at the University of Canberra were also presented to reinforce the ethical and responsible use of GenAI, particularly in relation to academic integrity. Finally, the session addressed the application of GenAI in completing the critical appraisal assessment, offering practical guidance on its appropriate use and highlighting common pitfalls to avoid. Collectively, this content laid the groundwork for students to develop both the technical competence and ethical awareness required for responsible GenAI use in their academic work.

##### 2.1.2.2 Online socialisation

We facilitated the tutorial by using Padlet (version 12.0.0), an interactive online platform that enabled students to engage in collaborative discussions and peer learning (34). This fosters online socialisation and cultivates a sense of community and shared learning. Students were invited to respond to a series of guiding questions designed to elicit both background perspectives and reflections on their evolving understanding of GenAI. The background questions encouraged students to share their initial experiences, expectations, and concerns:

1. How do you currently use (or hope to use) GenAI tools?
2. What are some of the current flaws or weaknesses of GenAI?
3. What would help you feel more confident using GenAI at university?

Following the hands-on demonstration of prompt strategies, students responded to evaluation questions such as:

- What differences did you notice after using the prompt strategies in the practical demonstration?
- How might you apply some of these strategies to enhance your own use of GenAI?
- Are there any additional concerns or drawbacks you would like to raise after participating in the tutorial?
- Final reflection and feedback: What are your takeaways from this session?

This shared online activity enabled students not only to reflect on their own learning but also to view and respond to the contributions of their peers. This interaction reinforced the principles of social constructivism, where knowledge is co-constructed through dialogue and collaboration (28). By establishing a safe, inclusive space for exchange, this stage played a crucial role in promoting engagement, confidence, and a sense of collective responsibility in using GenAI ethically and effectively.

##### 2.1.2.3 Information exchange

In this stage, students engaged in structured and guided activities to deepen their understanding of how GenAI can support academic tasks, particularly those involving the critical appraisal of public health literature. Over a two-hour tutorial session, students completed six hands-on activities designed to facilitate meaningful information exchange and application of learning. Students were guided in using Copilot to perform key academic tasks: summarizing scientific literature, identifying relevant references, and critically examining the design, results, and conclusions of published studies. These tasks mirrored the skills required in their critical appraisal assessment and were scaffolded to promote deeper engagement with both the content and the capabilities of GenAI tools.

A key focus of the tutorial was the introduction of the RTSI model, a structured approach to prompt writing that includes specifying the Role, Task, Structure, and Instructions (35). Students experimented with this model, generating outputs both before and after applying RTSI-based prompts. They then reflected on the changes in quality, relevance, and clarity of outputs through a collaborative activity on Padlet, allowing them to articulate what they had learned and observe patterns in their peers’ reflections. Further, students used Copilot to generate responses to specific questions from the critical appraisal assessment, guided by the Joanna Briggs Institute (JBI) tool for appraising randomized controlled trials (36). Importantly, students were tasked with critically engaging with the GenAI output, verifying statistics, and incorporating their own analytical insights based on what they had learned about evaluating peer-reviewed literature. This stage marked a transition from individual understanding to active application and collaborative learning, laying the groundwork for more independent and evaluative engagement with both GenAI and disciplinary knowledge.

##### 2.1.2.4 Knowledge construction

This stage focused on enabling students to independently construct new knowledge by integrating GenAI tools into the process of evidence appraisal and critique. Building on the foundational GenAI training and scaffolded learning activities from earlier stages, students were permitted to use Copilot to assist in completing the critical appraisal assessment. Students used Copilot to support tasks such as summarising study findings, evaluating methodological strengths and limitations, and interpreting statistical results. However, the emphasis at this stage was on assessing, verifying, and integrating Copilot responses with their own disciplinary knowledge and reasoning. To guide this process, students were encouraged to draw on epidemiological concepts learned in the first 10 weeks of the semester (37) and the JBI appraisal tool for randomised controlled trials. Academic staff provided ongoing support, helping students navigate common pitfalls such as overreliance on GenAI, misinterpretation of outputs, or failure to contextualise AI-generated content within public health evidence standards. This stage marked a transition toward more autonomous learning and knowledge generation. By constructing their own interpretations and judgments students demonstrated higher-order thinking, including analysis, synthesis, and evaluation. It also reinforced the understanding that GenAI is a complementary tool, not a substitute for academic rigor or critical reasoning. At the same time, students construct their knowledge of GenAI as a tool, its capabilities, limitations and the range of ethical considerations it presents.

##### 2.1.2.5 Development

The final stage of Salmon’s (32) model emphasises fostering independent, ethical, and reflective engagement with GenAI to support students’ ongoing academic and professional development. To address this need, students were required to submit a critical reflection on their use of GenAI during the completion of the critical appraisal assessment. This reflection accounted for 10% of the final grade, highlighting the importance of transparency, academic integrity, and self-awareness in the use of emerging technologies for academic assessments. To support this task, students were provided with a structured reflection framework that guided their analysis across five key dimensions:

1. Purposeful use of AI—how GenAI was used to support critical appraisal tasks such as identifying key arguments, summarising literature, or assessing evidence.
2. Critical thinking—evidence of critical engagement with GenAI outputs, including verification of claims and interpretation within the context of epidemiological principles.
3. Ethical and responsible use—consideration of ethical issues such as bias in AI-generated responses, transparency of use, and appropriate attribution.
4. Integration with personal analysis—the extent to which GenAI outputs were meaningfully balanced with the student’s own reasoning and disciplinary understanding.
5. Evaluation of outputs—assessment of the quality, credibility, and relevance of GenAI-generated content in supporting the task.

This reflective activity was designed to cultivate metacognitive awareness, encouraging students to think about what they learned, how they learned and applied GenAI in a principled and informed manner.

#### 2.1.3 Step 3: evaluation

This integration of GenAI into the Epidemiology and Principles of Research unit was evaluated using a convergent mixed-methods design. Data were collected using pre- and post-intervention surveys comprising both quantitative items and open-ended response questions. The survey instrument was developed in alignment with the study objectives and informed by existing literature on GenAI use in higher education (38). It was designed to capture key dimensions of students’ engagement with GenAI, including their knowledge, familiarity, perceived usefulness, confidence in using GenAI effectively, and attitudes toward its integration into learning and assessment. These constructs were measured using a combination of categorical and Likert-scale items. In addition, the survey included open-ended questions designed to elicit students’ reflective experiences, perceptions, and concerns regarding the use of GenAI. This combination of structured and open-ended items enabled both quantitative measurement and in-depth qualitative exploration of student experiences.

As part of the survey, students were invited to respond to the following open-ended questions: (1) Tell us about your overall experience with the integration of GenAI in this unit; (2) how might you apply some of the strategies you learned to enhance your own use of GenAI?; and (3) are there any concerns or drawbacks you would like to raise after completing the critical appraisal assessment using Copilot?. The responses provided qualitative insights into how students engaged with the technology, the challenges they encountered, and the reflective learning outcomes they experienced.

### 2.2 Participants and data collection

All students enrolled in the Epidemiology and Principles of Research postgraduate unit in 2025 (*n* = 110) were invited to participate in the study, constituting a census sampling approach. Participation was voluntary, and no exclusion criteria were applied. A total of 78 students completed the survey (Table 1), resulting in a response rate of 71%. As the study targeted a defined cohort within a single unit, no priori sample size calculation was performed. The achieved sample represents a substantial proportion of the eligible population, which is appropriate for exploratory mixed-methods research in educational settings. Ethical approval was obtained from the University of Canberra Human Research Ethics Committee (Approval number: 14600). Participants were provided with an information sheet on the survey cover page outlining the study purpose, procedures, and consent details. Informed consent was obtained electronically prior to survey completion.

**Table 1:** Pre-intervention survey responses showing participant demographics.

Variables	Frequency/percentage (%)
Age	
<25	30(38.5)
25–34	38(48.7)
35–44	6(7.7)
≥45	4(5.1)
Level of study	
Masters	74(94.9)
PhD	4(5.1)
Primary health discipline	
Occupational therapy	4(5.1)
Physiotherapy	26(33.3)
Public health	15(19.2)
Nutrition	3(3.8)
Pharmacy	2(2.6)
Speech pathology	26(33.3)
Other	2(2.6)
Year of study	
First	68(87.2)
Second	10(12.8)

A pre-GenAI survey was administered at the beginning of the semester, while a post-GenAI survey was conducted at the end of the semester, following the submission of students’ individual reflections. Survey data were collected using Qualtrics. The majority of participants were aged between 25 and 34 years (48.7%), followed by those under 25 years (38.5%). Most respondents were enrolled in a Master’s program (94.9%), with a small number pursuing PhD studies (5.1%). In terms of primary health discipline, the largest groups were from Physiotherapy (33.3%) and Speech Pathology (33.3%), followed by Public Health (19.2%). The majority (87.2%) of students were in their first year of study, with the remaining 12.8% in their second year.

### 2.3 Data analysis

Survey data were analysed using Stata 16. Descriptive statistics were first computed to examine frequency distributions across key variables. Fisher’s exact test was used to assess changes in student responses before and after the intervention. This test was selected due to the categorical nature of the variables and to account for small cell sizes in some response categories, where assumptions for chi-square tests may not be met. Following this, open ended responses which were de-identified and analysed using a thematic analysis approach, following the six-phase approach outlined by Braun and Clarke (39). NVivo software (version 15) facilitated line-by-line coding and visual organization of codes. An inductive approach (48) guided the analysis. Initial coding was conducted independently by ZM, followed by collaborative refinement with JE and JP. The research team engaged in iterative discussions to develop and finalize a shared codebook. Discrepancies were resolved through consensus, ensuring analytic rigor and consistency. Particular attention was paid to themes related to learning, ethics, digital literacy, and engagement with AI tools. Thematic categories were refined to ensure conceptual clarity and depth. Representative quotes were selected to illustrate and substantiate each theme. Finally, quantitative and qualitative findings were integrated during interpretation to provide a nuanced understanding of the views and experiences of students about the integration of GenAI into the Epidemiology ad Principles of Research unit.

## 3 Results

### 3.1 Generative AI knowledge, attitude and experience

Table 2 presents a comparison of participants’ knowledge, attitudes, and experiences with GenAI before and after the integration experience. A statistically significant improvement was observed in participants’ general understanding of GenAI following the intervention (*p* < 0.001), with the proportion of students reporting a moderate to extensive understanding increasing from 53.4 to 88.0%. Similarly, familiarity with using GenAI tools improved significantly (*p* < 0.001), as the combined proportion of students identifying as “familiar” or “very familiar” rose from 21.7 to 67.1%. In addition, there was a notable shift in participants’ self-reported ability to use GenAI effectively (*p* < 0.001). Prior to the intervention, only 25.0% of students considered themselves “effective” or “very effective” users, compared to 55.4% after the integrated experience.

**Table 2:** Generative AI knowledge and experience.

Variables	Pre GenAI frequency (%)	Post GenAI frequency (%)	Fisher’s exact test
General understanding of Generative AI			
No understanding	5(8.3)	1(1.3)	<0.001
Basic understanding	23(38.3)	8(10.7)	
Moderate understanding	25(41.7)	44(58.7)	
Advanced understanding	6(10.0)	15(20.0)	
Extensive understanding	1(1.7)	7(9.3)	
Familiarity with the use of Gen AI			
Very unfamiliar	5(8.3)	0(0.0)	<0.001
Not familiar	9(15.0)	0(0.0)	
Somewhat familiar	33(55.0)	24(32.9)	
Familiar	11(18.4)	38(52.0)	
Very familiar	2(3.3)	11(15.1)	
Find Gen AI useful			
Not useful at all	0(0.0)	0(0.0)	0.760
Not useful	1(2.9)	4(5.4)	
Somewhat useful	13(38.2)	22(29.7)	
Useful	12(35.3)	32(43.2)	
Very useful	8(23.6)	16(21.6)	
Find Gen AI easy to use			
Not easy at all	0(0.0)	0(0.0)	0.699
Not easy	1(2.9)	2(2.7)	
Somewhat easy	12(35.3)	33(44.6)	
Easy	16(47.1)	26(35.1)	
Very easy	5(14.7)	13(17.6)	
Abilities to use Gen AI effectively			
Not effective at all	8(13.3)	0(0.0)	<0.001
Not effective	11(18.4)	2(2.7)	
Somewhat effective	26(43.3)	31(41.9)	
Effective	12(20.0)	34(45.9)	
Very Effective	3(5.0)	7(9.5)	
Feeling about the integration of Gen AI			
Very negative	4(6.7)	0(0.0)	0.239
Negative	3(5.0)	3(4.1)	
Neutral	18(30.0)	20(27.0)	
Positive	24(40.0)	34(45.9)	
Very positive	11(18.3)	17(23.0)	
Believe that Gen AI can significantly improve epidemiological research			
Strongly disagree	2(3.4)	0(0.0)	0.753
Disagree	3(5.0)	3(4.3)	
Neutral	18(30.0)	22(31.4)	
Agree	26(43.3)	33(41.1)	
Strongly agree	11(18.3)	12(17.2)	
Interested in receiving more training or education on Gen AI			
Yes	49(83.0)	61(87.1)	0.002
No	10(17.0)	9(12.9)	
Have any concerns regarding the use of Gen AI			
Yes	26(43.3)	42(56.8)	<0.001
No	34(56.7)	32(43.2)	

Thematic analysis of the open-ended response data revealed six major themes: (i) tensions between automation and authentic learning, (ii) prompt literacy as a new academic skill, (iii) GenAI as a catalyst for metacognitive engagement, (iv) ethical ambiguity and cognitive dissonance, and (v) future-oriented learning. In the presentation of the thematic analysis below, we describe how students engaged with GenAI tools, perceived their educational value, and navigated the opportunities and challenges associated with their use. Illustrative quotes are included to highlight student voices, with each response assigned a participant number to ensure confidentiality.

### 3.2 Tensions between automation and authentic learning

Student responses revealed a perceived tension between the convenience of automation and the value of effortful engagement in learning tasks. While many participants acknowledged the potential of GenAI to simplify aspects of the assignment, others expressed concern that its use could limit deeper engagement with the material. This tension reflects a broader pedagogical challenge regarding how to integrate AI in ways that support critical thinking and independent reasoning. Several students described GenAI as requiring additional effort, particularly when outputs needed to be verified or corrected. One student shared:

*“I didn’t really use it as it felt more efficient to just collect the information myself.”* (ST34).

Similarly, another student highlighted the effort involved in reviewing AI-generated content:

*“It was more work to check and correct the AI’s mistakes than to just write the appraisal myself.”* (ST12).

These reflections suggest that for some students, the use of GenAI was perceived as increasing rather than reducing workload, particularly when outputs required critical evaluation. One student, reflecting on their experience with literature appraisal, noted:

*“Using AI to create draft points almost made more work for me than if I had just done the entire appraisal from scratch without AI making incorrect statements and misunderstanding results… It would have taken less brain power and time for me to do this without it.”* (ST06).

Other participants raised concerns about the alignment between assessment expectations and perceived real-world practices. For example, one student commented:

*“We were told specifically NOT to copy and paste from the AI, and to critically engage with the material. However, if we are to mimic what, presumably, will end up being real world use, people DO copy/paste from AI and just change a few things - it’s human nature, and AI is an amazing labour-saving device.” (ST71).*

Taken together, these accounts highlight students’ perceptions of a tension between efficiency and critical engagement when using GenAI. They also suggest variability in how students experienced the role of GenAI within the assessment task, particularly in relation to effort, verification, and expectations of appropriate use.

### 3.3 Prompt literacy as a new academic skill

Analysis of student responses revealed that the ability to formulate purposeful and context-specific prompts influenced student’s experiences of using GenAI. Students who reported difficulty in constructing prompts often described more limited usefulness of the tool, suggesting variability in how students engaged with GenAI. Several students reflected on the iterative nature of prompt formulation, highlighting that the quality of AI generated outputs appeared to be influenced by the clarity and specificity of their inputs. One student noted:

*“The way you ask the question really changes the answer. I had to experiment a lot to get anything useful.”* (ST28).

Another student expressed uncertainty about how to start interacting with GenAI:

*“Sometimes I didn’t even know what to ask to get the information I needed.”* (ST55).

This lack of confidence in prompt formulation was echoed by other participants, who indicated that GenAI’s usefulness was shaped by their ability to communicate effectively with the tool. For example, one student explained:

*“Gen AI’s outputs are only as good as the prompts you give it. It doesn’t replace humans as we are able to interpret the nuances. Also, fact checking Gen AI’s output is a must as it makes mistakes.”* (ST56).

Another student similarly reflected:

*“AI is extremely useful. It is on my part to improve on giving more effective prompts.”* (ST29).

These reflections suggest that students recognise the role of prompt formulation in shaping their interactions with GenAI, and that variability in prompt design influenced their experiences of the tool. Students’ reflections also indicate that engaging with GenAI required consideration of how to frame queries and evaluate the relevance and accuracy of outputs.

### 3.4 GenAI as a support for metacognitive engagement

Student reflections indicate that the use of GenAI was associated with increased awareness of their own thinking process. Several participants described engaging in reflection when comparing their own interpretations with AI-generated outputs, suggesting that GenAI may have prompted consideration of how they approached learning tasks. One student commented:

*“GenAI made me think more critically about the article I was reading instead of just summarising it*.*”* (ST7).

Another student described how the use of GenAI facilitated their engagement with complex content:

*“It helped break down complex concepts and pushed me to analyse more deeply.”* (ST28).

For some participants, this reflective engagement extended beyond content comprehension to include consideration of methodological and evaluative aspects of the task. One student explained:

*“Initially, I utilized GenAI to synthesize complex research articles… This approach enhanced my efficiency and allowed for more meaningful engagement in critical analysis rather than mere summarization. Through this process, I recognized the limitations of an over-reliance on AI-generated outputs… This highlighted the crucial role of human critical thinking in research appraisal, reinforcing the notion that GenAI should serve as an augmentative tool rather than an ultimate authority.”* (ST20).

Another student similarly noted changes in their approach to learning:

*“It made me think critically and differently.”* (ST38).

These accounts suggest that some students perceived GenAI as prompting reflection on their understanding, assumptions, and approaches to analysis. They also indicate variability in how students engaged with GenAI, particularly in relation to reflective and evaluative aspects of learning tasks.

### 3.5 Ethical ambiguity and cognitive dissonance

Student reflections revealed a sense of tension in their engagement with GenAI. While many participants acknowledged its usefulness in completing academic tasks, they also described concerns related to ethical considerations, specifically academic integrity and environmental impact. These responses suggest that students experienced uncertainty when navigating expectations around appropriate GenAI use. One student expressed concerns regarding the environmental implications of GenAI technologies:

*“As someone with major environmental concerns with generative AI, in that it requires substantially more energy and uses more water than a regular Google search, I am generally opposed to generative AI even in situations where it may be useful. I understand this is not a common belief though and respect the need to address this new frontier that is generative AI and educate students on its strengths and limitations.”* (ST19).

Other participants expressed concerns related to academic integrity, including plagiarism and the reliability of AI-generated content. For example, one student notes:

*“I was worried about plagiarism the whole time.”* (ST44).

Another highlighted uncertainty about the trustworthiness of AI outputs:

*“AI sometimes gives confident but wrong answers. It’s risky to rely on it.”* (ST07).

These responses suggest that students experienced ambiguity in relation to the ethical use of GenAI, particularly when balancing perceived efficiency with expectations of academic integrity. This uncertainty was further reflected in students’ descriptions of assessment requirements, as one student noted:

*“There were elements of the assignment that were confusing, and I felt very cautious and worried about plagiarism. But after the tutorial, I understand to use GenAI as the foundation of my ideas more than anything else.”* (ST41).

These responses suggest that students navigated multiple, and at times competing, considerations when using GenAI, including concerns related to academic expectations, reliability of outputs, and broader ethical implications.

### 3.6 Future oriented learning

Despite the challenges and limitations described, the majority of students reported that GenAI was relevant to their future professional practice and expressed appreciation for its inclusion in the Epidemiology and Principles of Research unit. They viewed the integration as aligned with evolving expectations in public health and research contexts.

One student reflected on the ethical and practical dimensions of learning to use GenAI:

*“I appreciated that we were taught how to use GenAI ethically. It’s something we’ll need in the workplace.”* (ST09).

Another participant highlighted the perceived relevance of the unit’s approach:

*“It felt modern and relevant, not just theoretical.”* (ST33).

This perception of relevance was further reflected in students’ broader views of technological change and professional preparedness:

*“Gen AI is changing the landscape, hence I believe it’s essential that we stay in the loop with regards to its new advancements. I’m glad we were introduced and had the opportunity to learn it in an ethical and modern fashion.”*

*“In today’s era of rapid advancements in Information Technology and Communication, Generative AI stands out as one of the most rapidly evolving technologies… Rather than avoiding its integration, it is imperative that we learn to utilise it responsibly and ethically.”*

These responses indicate that students perceived GenAI as having relevance to future professional contexts and valued opportunities to engage with the technology within an academic setting. They also suggest that students viewed structured exposure to GenAI as contributing to their understanding of its potential applications in practice.

## 4 Discussion

As GenAI continues to influence pedagogical practices in higher education, the integration of student perspectives is essential to fostering inclusive, ethical, and contextually responsive learning environments. The findings from this mixed methods evaluation reflect the complexity of integrating GenAI into tertiary education by combining quantitative evidence of change with qualitative insights into student experience. Students reported statistically significant improvements in their understanding of GenAI, familiarity with its use, and perceived ability to use it effectively following the intervention, suggesting increased confidence and capability. These quantitative findings are complemented by qualitative reflections which indicate how students experienced GenAI as influencing their engagement with knowledge, learning tasks, and their own cognitive processes. While student experiences were diverse, a consistent pattern across both datasets was the recognition that GenAI influenced how students approached learning in nuanced and context-dependent ways.

The results suggest that students experienced GenAI as a metacognitive tool which highlights its potential role in facilitating self-regulated learning. This is consistent with previous studies that position GenAI as supporting reflective and self-regulated learning processes in higher education (38, 40). However, this study extends existing work by demonstrating how structured integration within assessment tasks may prompt metacognitive engagement in discipline-specific contexts such as public health. As Zimmerman (41) suggests, metacognitive awareness is essential for students to plan, monitor, and evaluate their academic work. In this study, GenAI appeared to support such awareness by prompting students to reflect on their analytical processes, especially when comparing their reasoning with AI-generated outputs. However, the tension students described between automation and authentic learning reflects broader concerns about the use of educational technologies, consistent with Selwyn’s caution regarding the potential for superficial engagement (42). Some students expressed concern that GenAI could undermine critical thinking by offering prematurely summarised answers. These responses highlight perceived differences between independently conducted critical appraisal and GenAI-supported approaches, rather than demonstrating direct empirical comparisons between these methods. They also underscore the importance of scaffolding GenAI use in ways that support sustained critical engagement (33).

The emphasis on prompt literacy within student narratives reflects a shift in the type of competencies students perceive as important for engaging with AI tools. This finding is consistent with emerging literature identifying prompt literacy as an increasingly relevant capability in AI-mediated learning environments (49). Similar to earlier developments in digital literacy, prompt literacy may require students to engage more actively with how knowledge is generated and interpreted in interaction with AI systems (43). In this study, students described prompt formulation as both a technical and cognitive challenge, indicating the need for explicit instructional support within curricula (44).

The analysis also identified ethical ambiguity as a central part of the students’ experiences. Consistent with recent research (45), participants expressed concerns related to academic integrity and environmental implications of GenAI use. Their reflections suggest that students are actively engaging with questions of responsible AI use in academic contexts. The findings also indicate a degree of cognitive dissonance among students, as students navigated competing expectations related to efficiency, originality, and ethical responsibility. As Vučković and Sikimić (45) note, digital technologies introduce both cognitive and ethical considerations that require explicit attention within curriculum and pedagogy.

Finally, the theme of future-oriented learning suggests students’ interest in engaging with GenAI in ways that are professionally relevant. Students perceived the integration of GenAI as beneficial and aligned with future professional contexts in public health. In line with prior research (46), students viewed GenAI as relevant to their future practice. These findings suggest that embedding GenAI within discipline-specific assessments may contribute to students’ perceptions of preparedness for professional contexts, although this was not directly measured in the current study. This further supports calls for higher education to consider how emerging technologies are integrated into curricula to support both technical and ethical competencies (46).

This study has several strengths and limitations that should be noted. It explores student experiences of GenAI integration within a core public health unit, contributing to an area of growing interest in higher education. The findings provide insights into how students experience the use of GenAI in structured assessment contexts, which may inform ongoing discussions about its role in teaching and learning. However, this evaluation relied on survey data, including open-ended responses, and did not include structured baseline measures of students’ prior engagement with conventional critical appraisal tasks or their conceptions of authentic learning. This limits the ability to directly compare learning processes before and after the intervention. Future research should employ more in-depth qualitative approaches, such as focus group discussions or interviews, to provide a richer understanding of student experiences with GenAI use. Additionally, the inclusion of baseline and comparative measures would enable more robust evaluation of how GenAI integration relates to authentic learning in discipline-specific contexts.

## 5 Conclusion

The integration of GenAI into the Epidemiology and Principles of Research unit provided insights into how students engage with GenAI in ways that may support critical thinking and learning processes in postgraduate public health education. The findings suggest that embedding GenAI into teaching and assessment requires careful pedagogical design to support the development of competencies such as prompt literacy, critical engagement, and ethical reasoning. These competencies are relevant to academic development and professional practice in public health. The study also highlights the need for pedagogical approaches to consider the tensions between automation and authentic learning, as well as the ethical considerations associated with GenAI use, including its environmental implications. Addressing these issues may support the alignment of GenAI integration with principles of equity, academic integrity, and sustainability in higher education.

## Data Availability

The raw data supporting the conclusions of this article will be made available by the authors, without undue reservation.
